# Piperine Plays an Anti-Inflammatory Role in* Staphylococcus aureus* Endometritis by Inhibiting Activation of NF-*κ*B and MAPK Pathways in Mice

**DOI:** 10.1155/2016/8597208

**Published:** 2016-05-12

**Authors:** Wen-jun Zhai, Zhen-biao Zhang, Nian-nian Xu, Ying-fang Guo, Changwei Qiu, Cheng-ye Li, Gan-zhen Deng, Meng-yao Guo

**Affiliations:** College of Veterinary Medicine, Huazhong Agricultural University, Wuhan 430070, China

## Abstract

Endometritis is commonly caused by pathogenic microorganisms, including* Staphylococcus aureus* (*S. aureus*). Piperine, which is a natural medicine, has shown a variety of biological activities. To explore the effect and mechanism of piperine on* S. aureus* endometritis, a mouse model of* S. aureus* endometritis was successfully established in the present study. Histopathological changes were observed with H&E staining, cytokines were analyzed by ELISA, mRNA was analyzed by qPCR, and proteins were detected by western blot. The results showed that piperine could significantly alleviate inflammatory injury in* S. aureus* endometritis. The qPCR and ELISA results showed that piperine effectively reduced the* S. aureus*-induced overexpression of TNF-*α*, IL-1*β*, and IL-6 but increased the expression of IL-10. The* S. aureus*-induced inflammation was related to TLR-2 and TLR-4 because the results showed that their expression was increased in* S. aureus* infection but then decreased with piperine treatment. To further confirm that piperine caused an anti-inflammatory response by targeting NF-*κ*B and MAPKs, the expression of I-*κ*B, p65, p38, ERK, and JNK was measured. The phosphorylation of I-*κ*B, p65, p38, ERK, and JNK was inhibited by piperine in a dose-dependent manner. All of the results indicated that piperine may be a potential anti-inflammatory drug both in endometritis and in other* S. aureus*-induced diseases.

## 1. Introduction

Endometritis is a major disease in the postpartum period, and it affects both human and animal health [1, 2]. Endometritis can be caused by a variety of pathogens, including* Staphylococcus aureus* (*S. aureus*) [3] and* Escherichia coli* [4].* S. aureus*, which is a Gram-positive bacterium, is a common pathogen that causes purulent infections, such as pneumonia [5] and endometritis [3]. Many reports have shown that* S. aureus* induces inflammatory injury through TLRs and their downstream pathways, such as the NF-*κ*B and MAPK pathways [6, 7].

TLR-2 and TLR-4, which are members of the TLR family, are pivotal receptor proteins on the surface of cells mediating natural anti-infection immunity [8]. TLR-2 usually identifies the antigen on the surface of* S. aureus* [9], and some recent studies have shown that TLR-4 also plays a similar role [10]. NF-*κ*B and MAPKs are important molecules that should receive the signal transduced from the transformation of membrane receptors and, in turn, transmit it into the cell nucleus [11, 12]. TLR-2 and TLR-4 activation promotes the recruitment of adaptor proteins to activate NF-*κ*B [13], and some studies have indicated that MAPKs are also involved [14]. Activated NF-*κ*B and MAPKs stimulate the* S. aureus*-infected cells to produce a variety of inflammatory factors, such as the proinflammatory factors TNF-*α*, IL-1*β*, and IL-6 and the anti-inflammatory factor IL-10 [11].

Piperine, which is the major plant alkaloid from black pepper (*Piper nigrum*) and long pepper (*Piper longum*), has been reported to enhance bioavailability [15]. Previous studies have shown that piperine has many biological functions, including analgesic [16], anticonvulsant [17], antitumour [18], and anti-inflammatory [19] properties. The inflammatory reaction, a biological defence and repair mechanism of the innate immune system, is used to prevent harmful irritants, such as pathogens, from damaging cells and tissues. In the present study, an* S. aureus* endometritis model was successfully established in mice to explore the potential anti-inflammatory mechanism of piperine, which was not previously known.

## 2. Materials and Methods

### 2.1. Reagents

**Figure 1:**
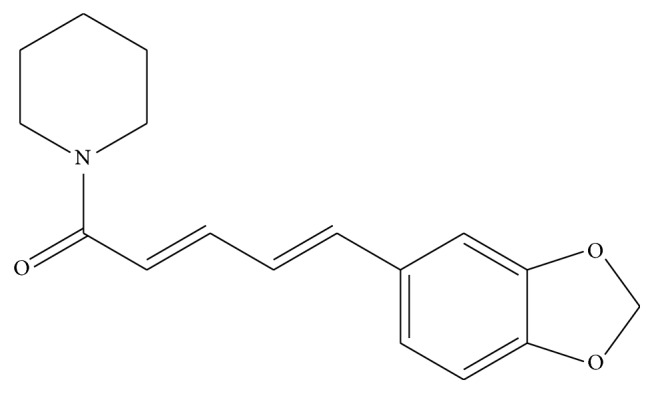
Chemical structure of piperine.

Piperine (purity: >98%, Figure 1) was purchased from the National Institute for the Control of Pharmaceutical and Biological Products (Beijing, China). TRIzol was obtained from Invitrogen. IL-1*β*, IL-6, IL-10, and TNF-*α* ELISA kits were acquired from BioLegend (Camino Santa Fe, CA, USA). All antibodies were purchased from Cell Signaling Technology (Beverly, MA, USA).* S. aureus* (ATCC 35556) was purchased from the American Type Culture Collection (ATCC, USA).

### 2.2. Animals and Experimental Groups

A total of 60 female BALB/c mice were used in this study. All of these mice were acquired from the Experimental Animal Center of Wuhan University (Wuhan, China). All experiments followed the guidelines for the care and use of laboratory animals published by the US National Institutes of Health. This study was approved by the Huazhong Agricultural University Animal Care and Use Committee. All mice were maintained on a 12 h light/dark cycle and cafeteria feeding.

Piperine was dissolved in 5 mL of tris buffered saline (TBS) at concentrations corresponding to 25, 50, and 100 mg/kg, based on the weight of the mice. After 24 h of* S. aureus* infection in the uterus, the piperine solution was injected intraperitoneally three times every 6 h. All mice were stochastically split into 4 groups as follows: (1) control group (CG), consisting of healthy mice that received no treatment; (2)* S. aureus* group (*S. aureus*), consisting of mice that received an injection of 100 *μ*L* S. aureus* in each side of the uterus horn and no drug treatment; (3) piperine treatment groups (PTGs), in which the* S. aureus*-infected mice were intraperitoneally injected with 25, 50, or 100 mg/kg of piperine; and (4) DEX group (DEX), in which the* S. aureus*-infected mice were cured by DEX. The mice were euthanized with sodium pentobarbital, and the uterus tissues were collected and stored at −80°C until analysis.

### 2.3. Histological Assays

The uterus tissues were gathered and fixed in 10% neutral buffered formalin. The uterus samples were embedded in paraffin, sliced, and stained with H&E reagent. After staining, the pathologic tissue sections were examined with a light microscope (Olympus, Japan).

### 2.4. Enzyme-Linked Immunosorbent Assays

The effects of piperine on the expression levels of* S. aureus*-induced inflammatory factors were examined in the uterus tissues. The uterus tissues were weighed and homogenized in phosphate buffered saline (PBS) on ice and then centrifuged at 12,000 rpm for 15 min at 4°C, and the supernatants were collected. The levels of TNF-*α*, IL-1*β*, IL-6, and IL-10 were detected by ELISA kits in the supernatants of uterus tissues, according to the manufacturer's directions. Each sample assay was repeated three times. The absorbance was read at 450 nm with a microplate reader (Thermo Scientific Multiskan MK3, USA).

### 2.5. Quantitative Real-Time Polymerase Chain Reaction (q-PCR) Assays

The total RNA was extracted from the uterus tissue samples of each group using TRIzol reagent according to the manufacturer's instructions (Invitrogen, China). The concentration and purity were determined spectrophotometrically at 260 and 280 nm. First-strand cDNA was synthesized using oligo. primers and Superscript II reverse transcriptase, according to the manufacturer's instructions (Invitrogen, USA). The synthesized cDNA was diluted five times with sterile water and stored at −80°C. The Primer Premier software (Premier Biosoft International, USA) was used to design specific primers for TNF-*α*, IL-1*β*, IL-6, IL-10, TLR2, TLR4, and GAPDH based on known sequences (Table 1). The qPCR was performed with an ABI PRISM 7500 Detection System (Applied Biosystems, USA). Reactions were performed in a 20 *μ*L reaction mixture containing 10 *μ*L of 2x SYBR Green I PCR Master Mix (TaKaRa, China), 1 *μ*L of diluted cDNA, 1 *μ*L of each primer (10 *μ*M), 0.8 *μ*L of 50x ROX Reference Dye II, and 6.2 *μ*L of PCR-grade water. The qPCR conditions were as follows: 95°C for 10 min, then 40 cycles of 95°C for 15 s, and 60°C for 60 s and 72°C for 60 s. The results (fold change) were expressed as 2^−ΔΔCt^. GAPDH was the reference gene. Each sample assay was repeated three times.

### 2.6. Western Blot Assays

The total protein was extracted from 100 mg of uterus tissues according to the manufacturer's recommended protocol (Invitrogen, Beijing, China). The BCA Protein Assay Kit was used to determine the protein concentrations. Samples with equal amounts of protein (50 *μ*g) were fractionated with 10% SDS polyacrylamide gels, transferred to polyvinylidene difluoride membranes, and blocked in 5% skim milk in TBST for 2 h. Then, the membranes were incubated with 1 : 1000 dilutions (v/v) of the primary antibodies at 4°C for 12 h. They were then incubated for 2 h with secondary antibodies diluted to 1 : 4000 (v/v). Densitometric values of immunoblot signals were developed with the ECL Plus Western Blotting Detection System (ImageQuant LAS 4000 mini, USA). Each sample assay was repeated three times. *β*-actin was used as a loading control.

### 2.7. Statistical Assays

Statistical analyses of the result data were performed using the SPSS software (ver. 15 for Windows; SPSS Inc., Chicago, IL, USA). All values are expressed as the mean ± SD. Significance was determined by a one-way ANOVA. A *p* value of < 0.05 was considered statistically significant.

## 3. Results

### 3.1. Histopathological Changes

**Figure 2:**
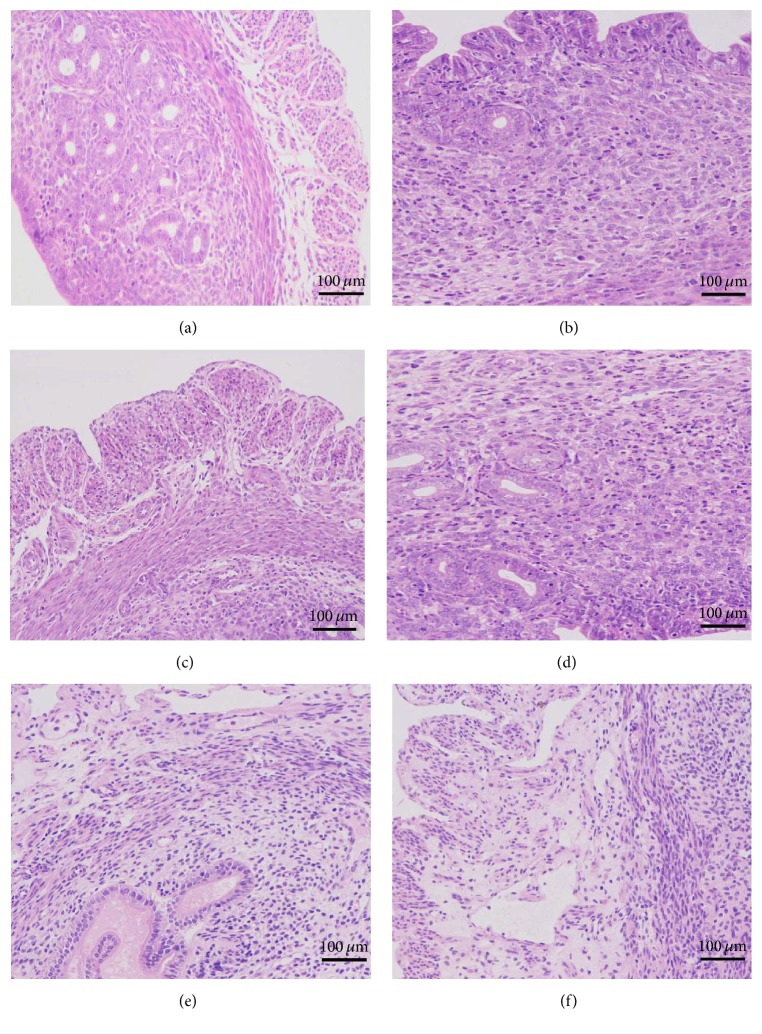
Histopathological changes of uterus tissues (H&E, ×200). (a) In the control group (CG), the mice received no treatment. (b) In the* S. aureus* group (*S. aureus*), the mouse model of* S. aureus* endometritis received no drug treatment. (c) In the DEX group (DEX), mice with* S. aureus* endometritis were treated with 5 mg/kg of dexamethasone. (d–f) In the piperine treatment groups (PTGs), mice with* S. aureus* endometritis were given 25 mg/kg, 50 mg/kg, or 100 mg/kg of piperine.

The inflammation injury could be directly seen with histopathological observation. The results are shown in Figure 2. No other uterine pathological changes were found in the CG (Figure 2(a)). In contrast to the CG, the* S. aureus*-treated samples showed injury (Figure 2(b)). The results showed that many of the epithelial cells were destroyed and that the hyperemia-related uterus tissues were infiltrated with a host of inflammatory cells, such as neutrophils and macrophages. These histopathological changes were ameliorated following treatment with piperine at doses of 25, 50, and 100 mg/kg (Figures 2(d)–2(f)).

### 3.2. Effects of Piperine on* S. aureus*-Induced Inflammatory Cytokines

**Figure 3:**
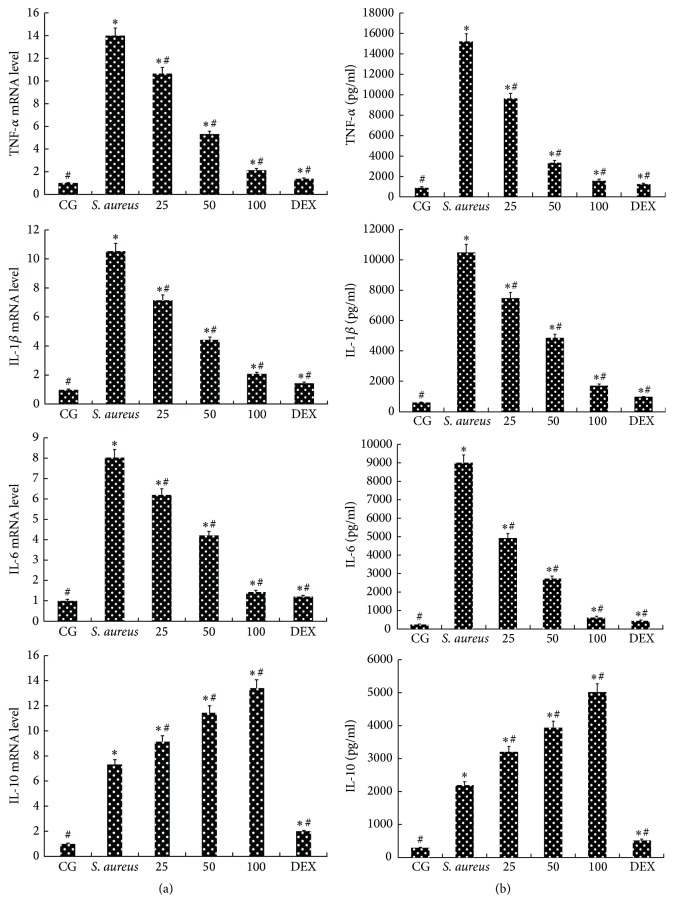
Expression of inflammatory cytokines. (a) The protein production levels of TNF-*α*, IL-1*β*, IL-6, and IL-10 in uterus tissues. (b) The mRNA levels of TNF-*α*, IL-1*β*, IL-6, and IL-10 in uterus tissues are shown. CG: control group.* S. aureus*:* S. aureus* group. The 25, 50, and 100 indicate the piperine treatment groups, which were given piperine at 25 mg/kg, 50 mg/kg, and 100 mg/kg per animal. DEX: DEX group, treated with 5 mg/kg. The data are represented as the mean ± SD. ^*∗*^ *p* < 0.05 represents statistical significance compared with the CG and ^#^ *p* < 0.05 represents statistical significance compared with the* S. aureus* group.

The ELISA and qPCR methods were used to analyze the expression of TNF-*α*, IL-1*β*, IL-6, and IL-10. The results are shown in Figure 3. The results showed that* S. aureus* apparently increased the expression of TNF-*α*, IL-1*β*, IL-6, and IL-10 compared with that in the CG. Compared with their expression in the* S. aureus* group, the expression of TNF-*α*, IL-1*β*, and IL-6 in the PTGs (25, 50, or 100 mg/kg) was clearly reduced in a dose-dependent manner (Figure 3(a)). However, the expression of IL-10 in the PTGs (25, 50, or 100 mg/kg) increased more than that in the* S. aureus* group in a dose-dependent manner (Figure 3(a)). The results showed that piperine can reduce the expression of proinflammatory factors (TNF-*α*, IL-1*β*, and IL-6), but it only improves the expression of anti-inflammatory cytokine IL-10. Furthermore, this effect became increasingly evident with an increasing piperine dosage (25, 50, or 100 mg/kg). The respective mRNA expression was consistent with the protein expression (Figure 3(b)).

### 3.3. Effects of Piperine on Expression of TLR-2 and TLR-4

**Figure 4:**
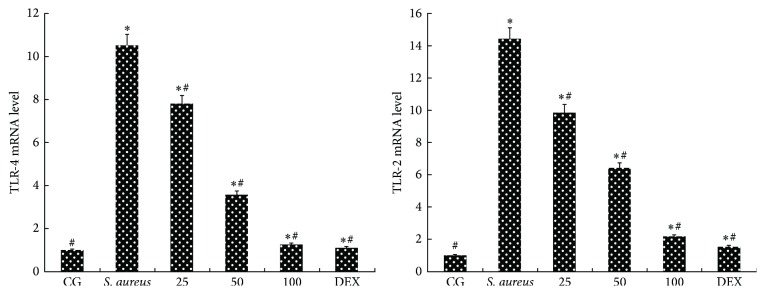
Effects of piperine on TLR-2 and TLR-4. The mRNA levels of TLR-2 and TLR-4 in uterus tissues. qPCR was performed to detect the TLR-2 and TLR-4 mRNA levels. GAPDH was used as a control. CG: control group, the mice without any treatment.* S. aureus*: the mouse model of* S. aureus* endometritis without any drug treatment. 25, 50, and 100: piperine treatment groups: the* S. aureus* endometritis mice were given piperine at 25 mg/kg, 50 mg/kg, and 100 mg/kg. DEX: the* S. aureus* endometritis mice were treated with dexamethasone at 5 mg/kg. The data are represented as the mean ± SD. ^*∗*^ *p* < 0.05 is significantly different from the CG; ^#^ *p* < 0.05 is significantly different from the* S. aureus* group.

TLR-2 and TLR-4 play key roles in* S. aureus*-mediated inflammatory responses [14]. The recognition of* S. aureus* by TLR-2 and TLR-4 can directly impact the activation of NF-*κ*B and MAPKs. The results showed that the mRNA levels of TLR-2 and TLR-4 increased significantly upon* S. aureus* infection. The* S. aureus*-induced increase was significantly (*p* < 0.05) suppressed by piperine in a dose-dependent manner (25, 50, or 100 mg/kg). The results are shown in Figure 4.

### 3.4. Effects of Piperine on the NF-*κ*B Pathway in* S. aureus*-Induced Endometritis

**Figure 5:**
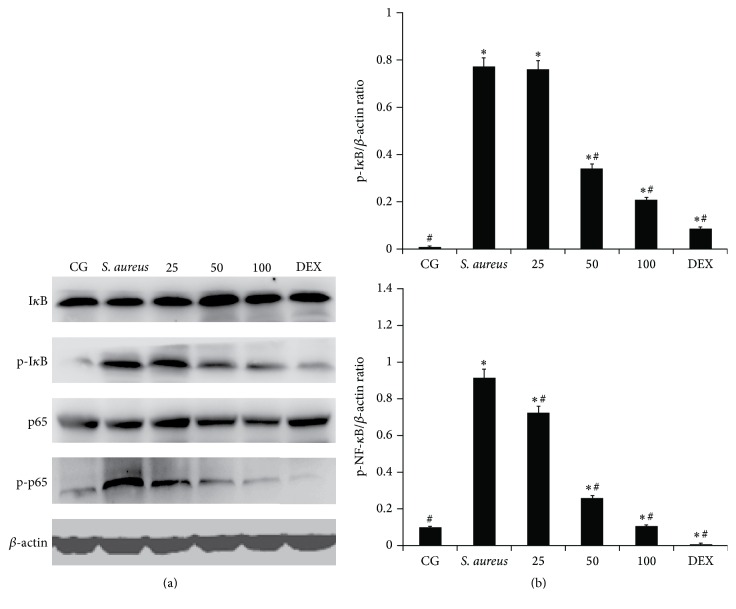
Effects of piperine on NF-*κ*B pathway activation. (a) The expression of NF-*κ*B p65 and I-*κ*B*α* is shown. (b) The ratio of NF-*κ*B p65 or I-*κ*B*α* and *β*-actin. CG: control group, the mice without any treatment.* S. aureus*: the mouse model of* S. aureus* endometritis without any drug treatment. 25, 50, and 100: piperine treatment groups: the* S. aureus* endometritis mice were given piperine at 25 mg/kg, 50 mg/kg, and 100 mg/kg. DEX: the* S. aureus* endometritis mice were treated with dexamethasone at 5 mg/kg. *β*-actin was used as a control. Values are presented as the means ± SD. ^*∗*^ *p* < 0.05 significantly compared with the CG; ^#^ *p* < 0.05 significantly compared with the* S. aureus*.

The NF-*κ*B signalling pathway is important for the inflammatory reaction of* S. aureus* infection [11]. To determine the effects of piperine, the NF-*κ*B activation was analyzed by western blot. The results showed that the phosphorylation of NF-*κ*B p65 and I-*κ*B*α* was significantly higher with* S. aureus*-infected mice than in the CG. The phosphorylated protein expression of NF-*κ*B p65 and I-*κ*B*α* was significantly inhibited by piperine treatment compared with their expression in* S. aureus*-infected mice that were not treated with piperine. With increasing concentrations of piperine (25, 50, and 100 mg/kg), the inhibition became more obvious, as shown in Figure 5.

### 3.5. Effects of Piperine on* S. aureus*-Induced MAPK Signalling Pathways

**Figure 6:**
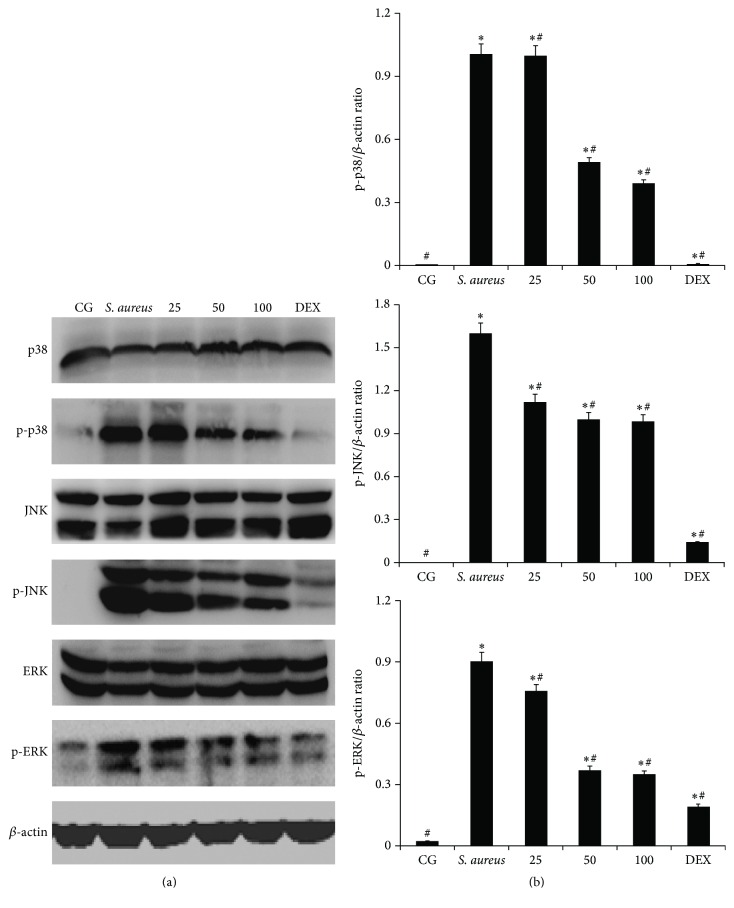
Effect of piperine on MAPK pathways activation. (a) The expression of p38, ERK, and JNK is shown. (b) The ratios of p38 or ERK or JNK with *β*-actin are shown. CG: control group, the mice without any treatment.* S. aureus*: the mouse model of* S. aureus* endometritis without any drug treatment. 25, 50, and 100: piperine treatment groups: the* S. aureus* endometritis mice were given piperine at 25 mg/kg, 50 mg/kg, and 100 mg/kg. DEX: the* S. aureus* endometritis mice were treated with dexamethasone at 5 mg/kg. *β*-actin was used as a control. ^*∗*^ *p* < 0.05 represents statistical significance compared with the CG and ^#^ *p* < 0.05 represents statistical significance compared with the* S. aureus* group.

MAPKs, which are activated by* S. aureus*, play significant roles in the development of endometrial inflammation [11]. To further determine the anti-inflammatory mechanism of piperine, the MAPKs (p38, ERK, and JNK) were examined by western blot, as shown in Figure 6. The phosphorylation of p38, ERK, and JNK was higher in* S. aureus*-stimulated uterus tissues. In the PTGs, the phosphorylation of p38, ERK, and JNK was significantly reduced compared with that in the* S. aureus* group. The results demonstrated that piperine can inhibit the phosphorylation of p38, ERK, and JNK in a dose-dependent manner (25, 50, or 100 mg/kg) in uterine tissues.

## 4. Discussion

Endometritis is a reproductive inflammatory disease in the postpartum period [20]. Many reports have shown that* S. aureus* can induce uterine inflammation [3, 21]. In the present study, a mouse model of* S. aureus* endometritis was successfully established.* S. aureus* injury was relieved by piperine, in agreement with the results of a previous study [22]. Histopathology changes showed that* S. aureus* induced serious injury to the uterus of mice, including nematode damage to the uterine structure and a decline in productivity and fertility [23].* S. aureus* infection increased the secretion of inflammatory cytokines, which is consistent with the findings of a previous study [24]. Piperine significantly inhibited the* S. aureus*-induced excessive secretion of proinflammatory factors (TNF-*α*, IL-1*β*, and IL-6) but increased the secretion of an anti-inflammatory factor (IL-10). Additionally, these inflammatory responses were controlled by piperine in a dose-dependent manner.

TNF-*α* and IL-1*β*, which are typical primary cytokines, play a role in initiating the inflammatory response [25]. TNF-*α* induces the infiltration and activation of neutrophils and enhances the expression of cellular adhesion molecules, but it impairs endothelial cells and aggravates the cascade of other inflammatory mediators [26, 27]. The expression of TNF-*α* was reduced after piperine treatment, which indicates that piperine could alleviate inflammation by inhibiting the effects of neutrophils on the cells in the endothelial monolayer [28]. IL-1*β*, which is a major catabolic factor, is key in the regulation of host immune responses against* S. aureus* [29, 30]. The decrease in IL-1*β* expression proved that piperine could resist inflammation. IL-6 plays an essential role in neutrophilic inflammation [31]. IL-6, which is promptly and transiently produced in infections and tissue injury responses, contributes to the host defence through the stimulation of acute-phase responses, hematopoiesis, and immune reactions [32]. The decrease in IL-6 expression indicated that piperine had an anti-inflammatory effect. In contrast, IL-10 may be necessary to reduce collateral injury due to inflammatory responses [33]. IL-10, which is a multifunctional cytokine, plays a pivotal role in suppressing the production of proinflammatory mediators [34]. The increase in the IL-10 expression level meant that piperine could play an anti-inflammatory role. TNF-*α*, IL-1*β*, IL-6, and IL-10 have been implicated in the initiation and regulation of the immune response and inflammation [14, 35, 36]. In summary, piperine showed anti-inflammatory effects by decreasing the overexpression of proinflammatory factors (TNF-*α*, IL-1*β*, and IL-6) and increasing the expression of an anti-inflammatory factor (IL-10). Additionally, the results of the present study are in agreement with those of previous studies [37, 38].

*S. aureus* is identified mainly through TLR-2 [24], but some studies have confirmed that TLR-4 also plays an important role in the process of* S. aureus*-induced inflammation [10]. The stimulation of TLR-2 and TLR-4 can lead to the production of different cytokines [14]. TLR-2 and TLR-4 activated downstream pathways to improve the expression of inflammatory factors (TNF-*α*, IL-1*β*, IL-6, and IL-10). Many studies have shown that the excessive expression of TLR-2 and TLR-4 promoted an active inflammatory reaction [14, 39]; the reduction of TLR-2 and TLR-4 expression in this study confirmed that piperine could inhibit inflammation.

To further probe the anti-inflammatory mechanism of piperine, the expression of NF-*κ*B (NF-*κ*B p65 and I-*κ*B*α*) and MAPKs (p38, ERK, and JNK) was examined by western blot. NF-*κ*B is a ubiquitous heterodimeric transcription factor that exists in an inactive form in the cytoplasm, bound to the inhibitory proteins referred to as I-*κ*B [40]. The NF-*κ*B pathway has been shown to be involved in numerous inflammatory diseases [2, 11]. The suppression of NF-*κ*B p65 and I-*κ*B*α* showed the anti-inflammatory effect of piperine. MAPKs are part of another pathway in the regulation of inflammatory responses and include the proteins p38, ERK, and JNK [12]. In the present study, the phosphorylation of p38, ERK, and JNK was significantly decreased by piperine. This showed that piperine played an anti-inflammation role by inhibiting the expression of p38, ERK, and JNK, and these results were consistent with those from previous studies [41, 42].

## 5. Conclusion

In conclusion, piperine showed an anti-inflammatory effect in* S. aureus*-induced endometritis. Piperine inhibited the expression of TLR-2 and TLR-4 and the activation of the NF-*κ*B and MAPKs pathways, thereby preventing the excessive secretion of proinflammatory factors (TNF-*α*, IL-1*β*, and IL-6) and increasing the secretion of an anti-inflammatory factor (IL-10). After further research, piperine may be an effective drug for the clinical therapy of* S. aureus* endometritis and other infections.

## Figures and Tables

**Table 1:** Sequence of primers used for qPCR assay.

Gene	Accession number	Primers	Product
IL-1*β*	NM_008361.3	Sense: 5′-AGGCTCCGAGATGAACAA-3′	264 bp
		Antisense: 5′-AAGGCATTAGAAACAGTCC-3′	
TNF-*α*	NM_013693.2	Sense: 5′-CTTCTCATTCCTGCTTGTG-3′	198 bp
		Antisense: 5′-ACTTGGTGGTTTGCTACG-3′	
IL-6	NM_031168.1	Sense: 5′-TTCTTGGGACTGATGCTG-3′	180 bp
		Antisense: 5′-CTGGCTTTGTCTTTCTTGTT-3′	
TLR-2	NM_011905.3	Sense: 5′-TTTGCTCCTGCGAACTCC-3′	267 bp
		Antisense: 5′-GCCACGCCCACATCATTC-3′	
TLR-4	NM_021297.2	Sense: 5′-TTCAGAGCCGTTGGTGTATC-3′	170 bp
		Antisense: 5′-CTCCCATTCCAGGTAGGTGT-3′	
GAPDH	NM_008084.2	Sense: 5′-AGGTCGGTGTGAACGGATTTG-3′	195 bp
		Antisense: 5′-GGGGTCGTTGATGGCAACA-3′	
